# First records of *Dermacentor albipictus* larvae collected by flagging in Yukon, Canada

**DOI:** 10.1186/s13071-020-04425-3

**Published:** 2020-11-11

**Authors:** Emily S. Chenery, N. Jane Harms, Nicholas E. Mandrak, Péter K. Molnár

**Affiliations:** 1 Department of Physical and Environmental Sciences, University of Toronto Scarborough, 1265 Military Trail, Scarborough, Ontario M1C 1A4 Canada; 2 Animal Health Unit, Environment Yukon, 10 Burns Road, Whitehorse, Yukon Y1A 4Y9 Canada; 3 Department of Biological Sciences, University of Toronto Scarborough, 1265 Military Trail, Scarborough, Ontario M1C 1A4 Canada

**Keywords:** *Dermacentor albipictus*, Flagging method, Larval tick, Winter tick, Yukon, Canada

## Abstract

**Background:**

The winter tick (*Dermacentor albipictus*) has garnered significant attention throughout North America for its impact on wildlife health, and especially for moose (*Alces alces*), where high tick burdens may result in host hair loss, anemia, and can prove fatal. The environmental transmission of *D. albipictus* larvae to a host is a critical event that has direct impact on infestation success, yet in-field observations of this life stage are lacking. In Yukon, Canada, *D. albipictus* had previously been found on hosts, but its larval life stage had not been detected in the field, despite previous sampling attempts.

**Methods:**

We sampled for *D. albipictus* larvae using traditional flagging methods in Ibex Valley and Braeburn, Yukon. Sites were sampled repeatedly for *D. albipictus* larvae by flagging from late August to end of October in 2018 and late August to end of November 2019.

**Results:**

Larvae of *D. albipictus* were collected throughout Ibex Valley, at approximate densities ranging from 0.04 to 4236 larvae/100 m^2^. Larvae were present primarily on grassy vegetation on south-facing slopes in the Ibex Valley region and in Braeburn. Highest average larval numbers suggest peak questing activity was towards the end of September and beginning of October, as elsewhere in North America.

**Conclusions:**

To the best of our knowledge, we report the first successful collection of the off-host, larval life stage of *D. albipictus* by flagging, north of 60° latitude in Yukon, Canada. These new observations provide critical information on the spatial distribution of the host-seeking life stage of *D. albipictus* and confirm that this species is completing its whole life cycle in southern Yukon. Understanding the environmental conditions where larvae spend their vulnerable period off-host in this northern location can inform both management strategies and projections of future range expansion which may occur with a changing climate. 
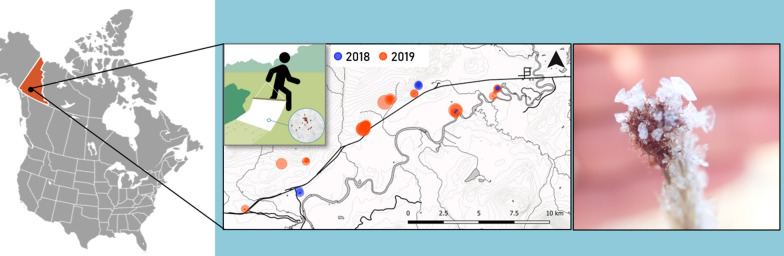

## Background

Understanding the off-host dynamics of tick life-cycles has profound implications for designing successful surveillance programmes [1], predicting future risk to humans and wildlife [2, 3], and determining appropriate management options [4]. Prior to our study, collection of the off-host, larval life stage of the winter tick, *Dermacentor albipictus* (Packard, 1869) had not been documented at what is believed to be one of the northernmost limits of their range, in Yukon Territory, Canada [5–7].

*Dermacentor albipictus* is a one-host ixodid tick with a one-year life-cycle, which primarily feeds on cervids such as moose (*Alces alces*), elk (*Cervus canadensis*), caribou (*Rangifer tarandus*), and deer (*Odocoileus* sp.). Although it shows no host specificity, infestation in moose is well-known for causing significant hair and blood loss, the effects of which can be fatal [6, 8, 9]. Winter ticks have also been implicated in the death of elk, showing similar pathological features [10]. It is not known to be a vector of any significant diseases of public or wildlife health concern, although ongoing research suggests *D. albipictus* may be a reservoir for some pathogens, such as pseudorabies [11] and *Babesia duncani* [12].

The initial introduction of *D. albipictus* to Yukon was likely through the translocation of elk from Elk Island National Park, Alberta, Canada, in the 1950s and 1990s [13–15]. Prior to this, the ticks were not believed to be present north of 60°N latitude [5, 6] though likely present up to 64°N latitude in the adjacent Northwest Territories [5, 13, 16]. Samuel’s comprehensive survey of trappers in northwestern Canada in 1987 indicated that, anecdotally, some Yukon moose may have had hair loss indicative of *D. albipictus* infestation as far back as the 1930s [13], but no field studies had been otherwise conducted. Monitoring by the Yukon government has recorded nymphal and adult *D. albipictus* by examining cervid hides since 2012, but no larval ticks had been detected in the environment, despite multiple flagging attempts 2010–2012 [7, 15].

Here, we report for the first time the collection of larval *D. albipictus*, by flagging in 2018 and 2019, in important cervid habitat in Yukon, Canada, thus confirming that winter ticks are successfully completing their life-cycles despite the high latitude.

## Methods

**Fig. 1:**
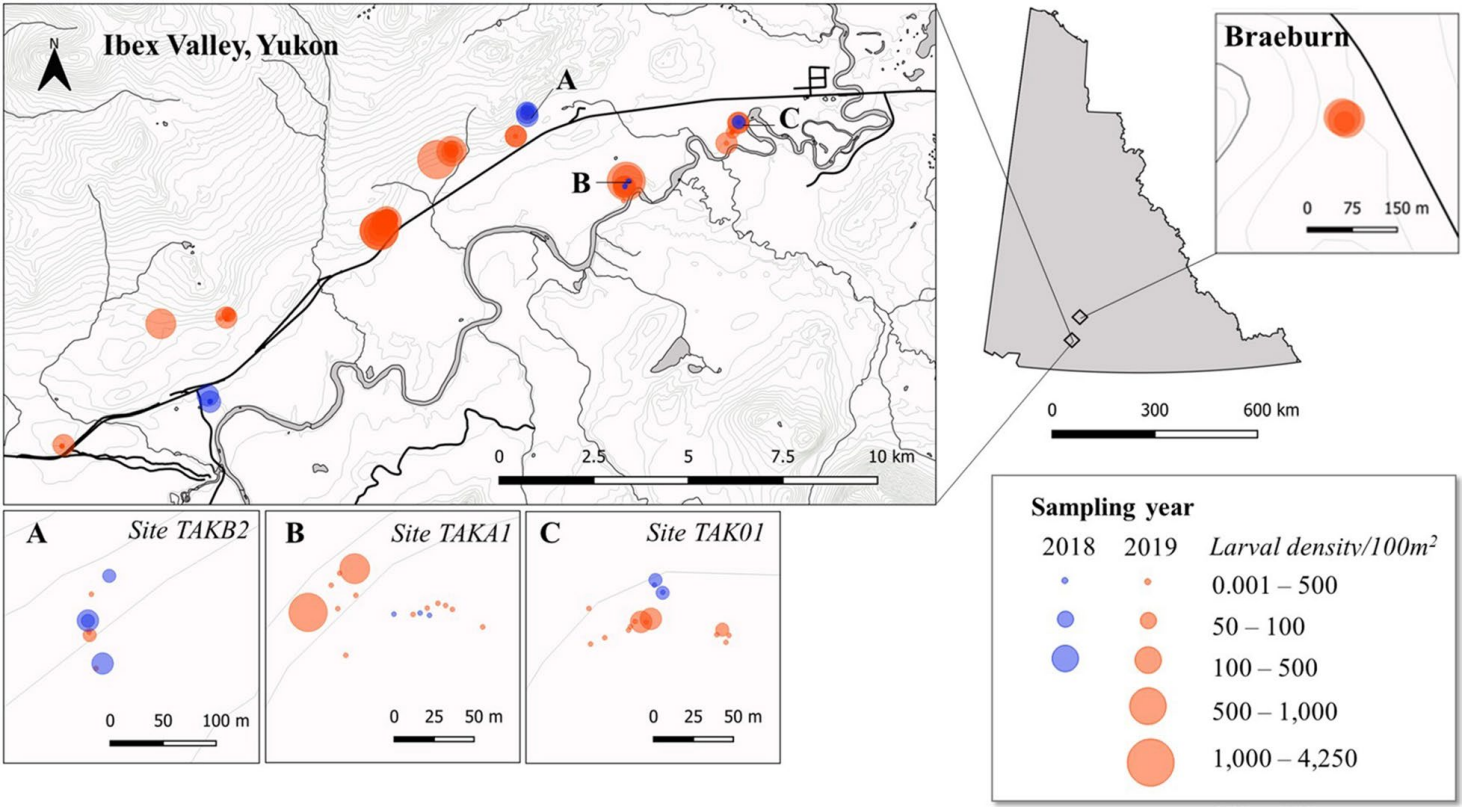
Locations of larval tick sampling sites in 2018 (blue) and 2019 (orange) in Ibex Valley and Braeburn, Yukon, Canada, either side of the Alaska Highway (black line). Bubble size indicates the average densities of *D. albipictus* larvae collected per 100 m^2^. Highest larval densities sampled were in the central region of Ibex Valley, and at Braeburn. Larvae were often detected at almost identical locations in 2019 as the previous year (inset: **a**, **b**) or at very close proximity to these locations (inset: **c**)

The Ibex Valley is located in the Boreal Cordillera ecoregion of southern Yukon, Canada (60°50′42″N, 135°38′18″W, elevation *c.*721 m), approximately 16 km west of the city of Whitehorse (Fig. 1). It is primarily settlement land of the Champagne and Aishihik and Kwanlin Dün First Nations and is presently undeveloped, with a few agricultural holdings of livestock and private residences. The approximately 152 km^2^ area also forms the core range for a managed population of elk (*Cervus canadensis*) that moved into this region following their introduction in 1959 [15, 17]. Other potential host species found here include moose, mule deer (*Odocoileus hemionus*), and semi-feral horses (*Equus caballus*). Vegetation composition is mixed, with characteristic boreal forests of conifers mixed with wetlands and aspen stands, interspersed with dry, grassy south-facing slopes and glacial lacustrine valley bottoms [18]. Several areas have shown slow regeneration since forest fires in 1958 [18] and are sparsely vegetated.

The Ibex Valley formed part of a pilot study in 2018 aimed at obtaining an initial detection of *D. albipictus* larval presence, and it was the only location where any larvae were found (Fig. 1). In 2019, all sampling efforts focused on this region with the aim of confirming larval detections made the previous year. Twelve sites, each a minimum of 500 m^2^ were selected across the valley based on habitat type (sub-polar grassland, barren-ground) and host activity (game trails, pellets, tracks). One additional site was also chosen in the Braeburn elk range (61°25'20.2"N, 135°43'52.1"W, elevation *c.*835 m), 40 km to the north of Ibex Valley. Flagging was conducted at each site, focussing on areas with visible cervid game trails. Sampling events were repeated, at minimum, every two weeks from 26 August to 30 November 2019 during daylight hours and did not take place on days of heavy rain or high winds but continued after snowfall (8 October 2019 onwards). Low densities of larval ticks were immediately removed from flag samplers in the field using lint rollers, and lint sheets were placed in sealed plastic bags. Flags with high larval abundances were removed and placed directly in sealed bags. All lint and flag samples were labelled in the field and returned to the lab for identification and counting. Where possible, questing vegetation was identified to family or species level in the field, consistent with Cody (2000) [19]. Vouchers and photographs were collected of any species not positively identified in the field and were later identified with the assistance of a Yukon Government expert (B. Bennett, pers. comm*.*, 26/09/19). Local temperature and relative humidity data were collected for each sampling location using a Kestrel environmental meter (Kestrel 5000 handheld Environmental Meter, Nielsen-Kellerman PA, USA). In both sampling years, tick identification was carried out *via* microscopy on a subset of each sample, based on morphological characteristics provided in Lindquist et al. and as reported in Clifford et al. [20, 21]. Additionally, in 2018, several specimens were preserved in 70% ethanol and submitted for confirmatory identification (Canadian Science Centre for Human and Animal Health, Winnipeg, Manitoba, Canada). All larvae were confirmed to be *D. albipictus* and no other tick species were detected.

## Results and discussion

**Fig. 2:**
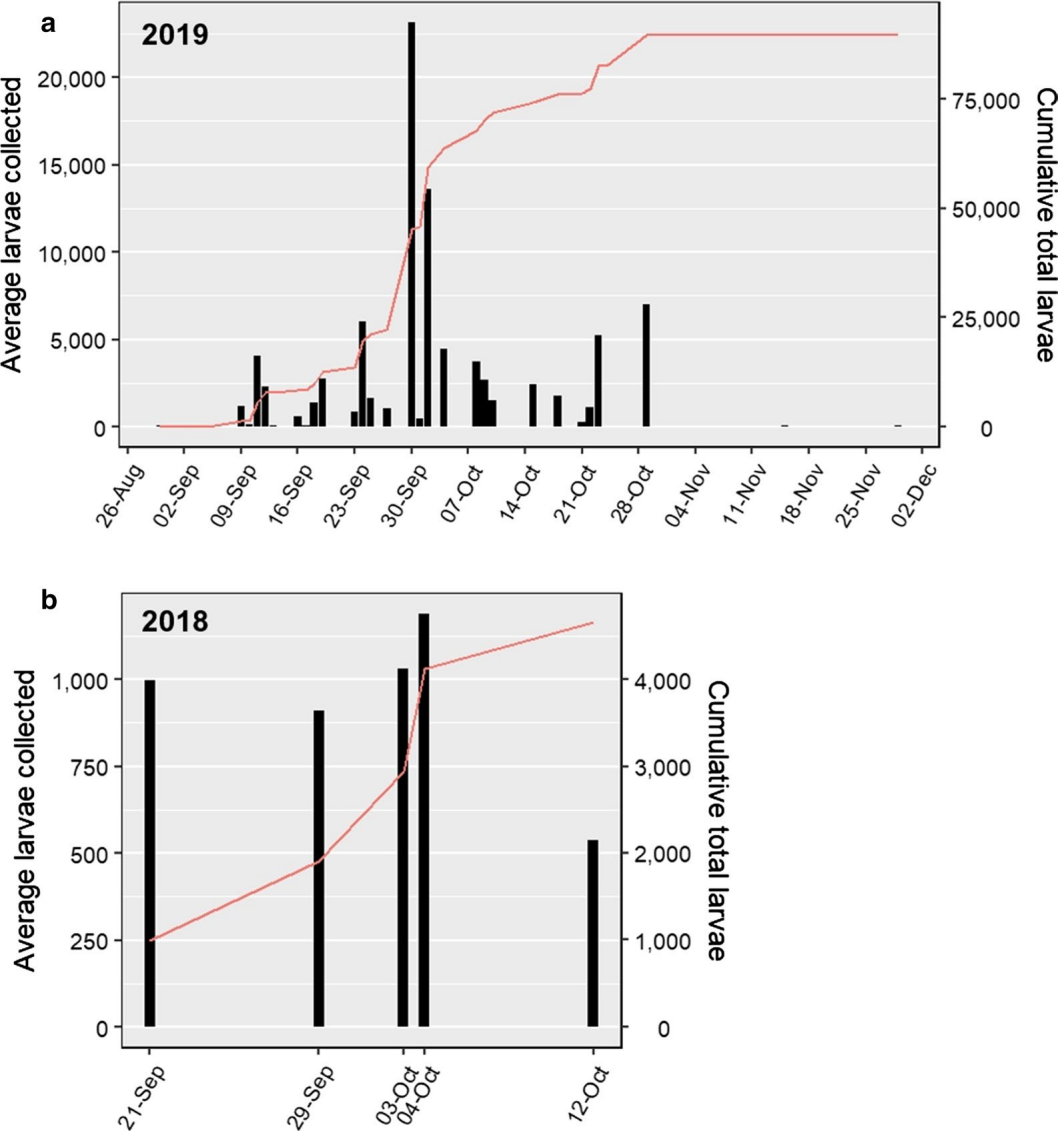
Average number of *D. albipictus* larvae collected per sampling day (black bars) and cumulative total (red line) across all sites sampled in Ibex Valley, 2019 (**a**) and 2018 field seasons (**b**). Although two weeks of sampling were missed end of October-beginning of November 2019, both average daily collection numbers and cumulative total suggest that peak questing most likely occurred during the last week of September and first week of October

A cumulative total of 6,924 *D. albipictus* larvae were collected across Ibex Valley in 2018 (21 September–18 October), and 135,582 in 2019 (30 August–30 November). Approximate densities ranged from 0.22–146.2 larvae per 100 m^2^ in 2018, and from 0.044 larvae per 100 m^2^ in 2019 (Figs. 1, 2). The difference, in both detection periods and approximate numbers and densities of ticks per season, is likely due to our increased knowledge of suitable sampling locations and associated efforts in 2019, rather than a reflection of actual tick activity each year. A total of 7238 *D. albipictus* larvae, ranging 184.8–3293.7 larvae per 100 m^2^, were also collected in Braeburn in 2019 during two sampling events (19 September and 4 October). Only sites in Ibex Valley were sampled until 30 November 2019, but it seems plausible that larvae continue actively questing at all previous tick detection locations until at least this date. In all but one of the locations where larvae were found in 2018, larvae were also present in 2019, often at almost identical points (Fig. 1: insets A, B), or in extremely close proximity to the previous sampling points (Fig. 1: inset C). This finding suggests a high degree of site fidelity among cervid and equine hosts may result in spatial ‘hotspots’ of larvae that are consistent year-to-year.

**Fig. 3:**
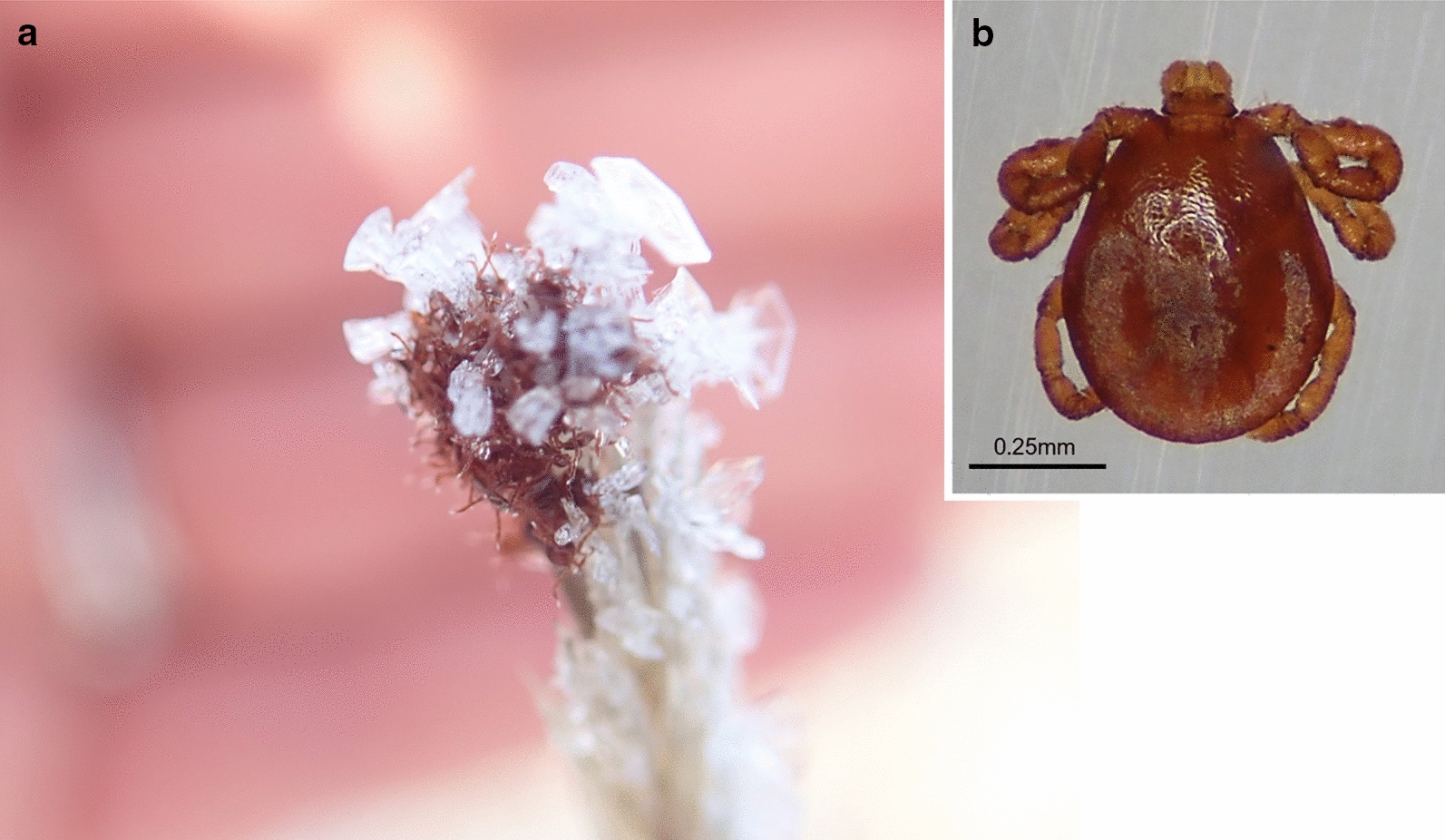
**a** Larvae of *D. albipictus* actively questing beneath ice crystals, Ibex Valley, Yukon, Canada, on 21 October 2019. Once contact was made, these larvae were still capable of attaching to a flag sheet, suggesting that relatively extreme conditions in early winter may not significantly reduce transmission to a host during this period. **b** Magnified dorsal view of *D. albipictus* larva. (Image credits: E.S. Chenery)

We observed larvae actively questing from 30 August to 29 November 2019. On average, the number of larvae collected per day across all tick-positive sampling sites was highest during the last week of September and first week of October (Fig. 2). Due to the limited data available for 2018, conclusions regarding questing peak for that year cannot be drawn. This apparent peak in questing activity observed in 2019 is similar to the reported behaviour for *D. albipictus* elsewhere in North America [22–26], and a lack of difference in the north may indicate that questing is not solely driven by temperature, as has been previously suggested [5, 24–26]. Temperature and relative humidity measured on site over this sampling period varied considerably, from − 2.3 to 33.5 °C, and 15.1–91.5%, respectively (Table 1). Field temperatures were generally warmer than the daily reported averages by 5–19 °C, reinforcing the value of field measures in capturing microhabitat conditions influential for tick survival and development at this high latitude (Table 1). Critically, previous laboratory experiments of the cold tolerance of *D. albipictus* from New Hampshire, USA, have suggested unlikely survival of larvae after contact with ice [27], yet we frequently observed active larvae under these conditions in the field in Yukon (Fig. 3). Previous genetic analysis of a sample of Yukon *D. albipictus* adults suggested that this population is most likely a combination of translocated ticks from Elk Island National Park in Alberta, Canada, and *D. albipictus* that have expanded their range from nearby northern British Columbia [14]. Early experiments have also shown that adult females are capable of egg-laying in adjacent Alaska [28], suggesting that environment and habitat may not be as critical a limiting factor in this species' distribution, as once thought [5, 6, 15]. In absence of any clear genetic differences, however, we might hypothesise that phenotypic changes may have arisen in this Yukon population in the decades since their first arrival. Given this, and the apparent discrepancy between our observations and those of *D. albipictus* survival in more southerly regions (e.g. [27]), further comparisons between northern and southern populations of *D. albipictus* larvae may be warranted to determine if there are significant differences in their ability to tolerate environmental extremes across latitudes.

**Table 1:** Averaged weekly measurements of temperature and relative humidity, collected in the field across sampling locations in 2019, Ibex Valley, Yukon, Canada

Sampling week, 2019		Temperature (°C)			5-day mean	Relative humidity (%)		
		Min	Mean	Max		Min	Mean	Max
1	19–24 August	–	–	–	7.9	–	–	–
2	26–31 August	25.0	27.8	33.5	8.8	23.9	25.1	25.7
3	2–07 September	23.0	23.4	25.0	18.2	29.5	35.7	44.8
4	9–14 September	16.7	21.2	29.7	8.8	15.6	34.8	59.6
5	16–21 September	13.7	17.3	20.1	10.2	39.9	51.1	69.2
6	23–27 September	12.0	16.1	22.6	5.4	25.5	33.2	59.0
7	30 September–5 October	8.0	15.0	19.5	–	34.6	54.6	91.5
8	7–12 October	− 2.3	5.8	9.7	–	46.4	56.1	67.7
9	14–19 October	0.9	1.8	3.6	–	33.0	50.4	85.3
10	21–26 October	3.6	4.8	5.6	− 2.6	–	52.0	–
11	28 October–2 November	6.1	6.2	6.3	− 0.5	–	56.2	–
12	4–9 November	–	–	–	− 7.7	–	–	–
13	11–16 November	− 0.6	2.5	6.0	− 7.7	69.6	70.5	71.3
14	18–23 November	4.6	5.2	5.8	− 0.4	50.1	55.6	61.1
15	25–30 November	–	− 0.5	–	− 10.7	–	58.3	–

*Notes*: Five-day mean values for temperature were calculated from daily averages reported at the nearest meteorological station (Takhini River Ridge: 60°56'45.0"N, 135°34'23.0"W, elevation 671 m) [33]. Mean values for relative humidity were not available. Missing values are indicated by dashes and include weeks one and 12 when sampling was absent

**Fig. 4:**
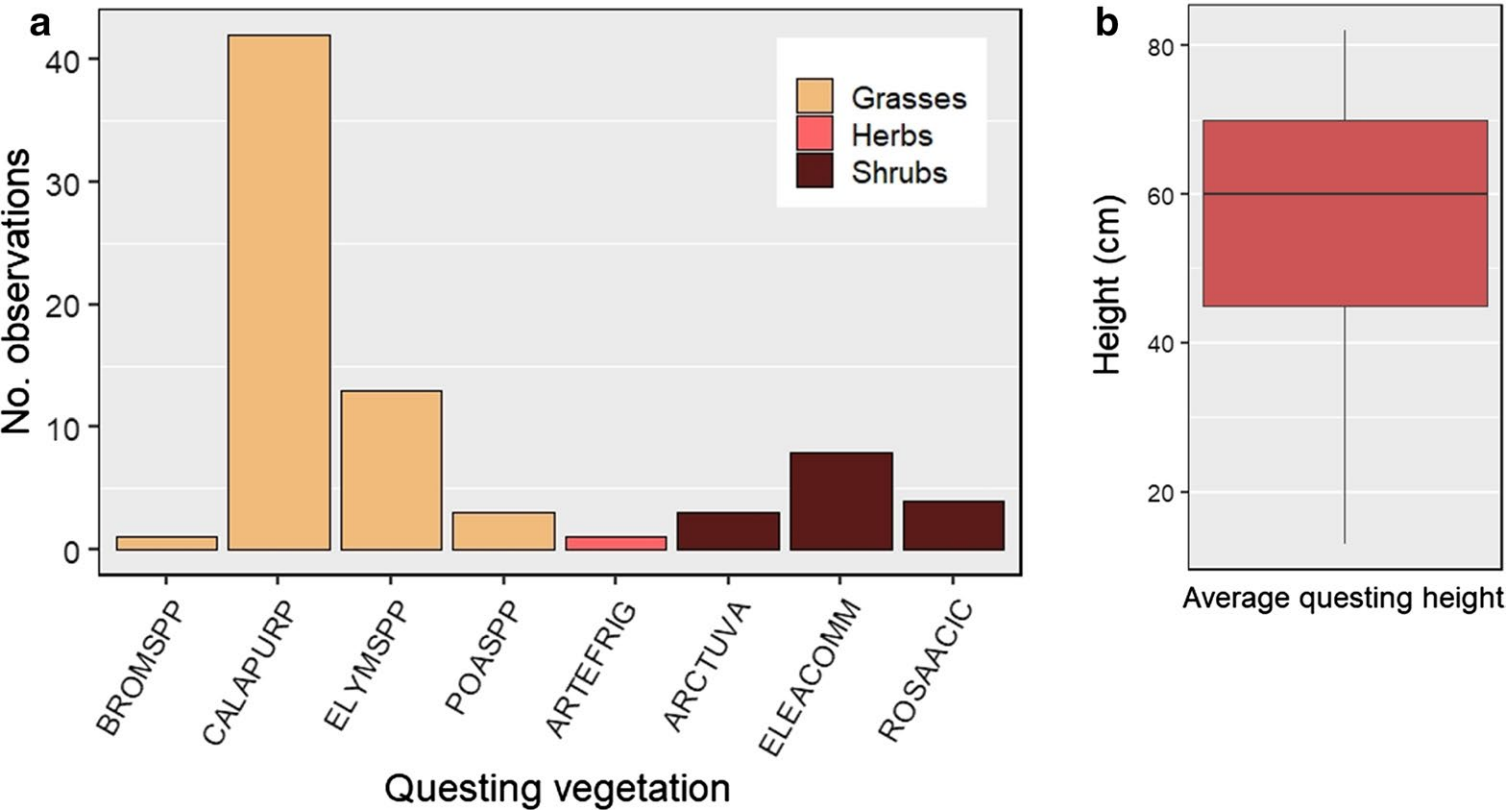
**a** Vegetation species and groups upon which larval *D. albipictus* were found questing in 2018 and 2019. Species codes are as follows: (Grasses) BROMSPP = *Bromus* sp., CALAPURP = *Calamagrostis purpurea*; ELYMSPP = *Elymus* sp., POASPP = *Poa* sp.; (Herbs) ARTEFRIG = *Artemisia frigida*; (Shrubs) ARCTUVA = *Arctostaphylos uva-ursi*, ELEACOMM = *Eleagnus commutata*, ROSAACIC = *Rosa acicularis.* **b** Average height of questing aggregations, 2018 and 2019. In almost all cases, questing height was identical to the observed maximum height of the vegetation available

Most questing aggregations were observed on grasses, particularly *Calamagrostis purpurea,* however, larvae were also found on other vegetation where it was available (Fig. 4a). No detections were made in coniferous forest or closed canopy areas, consistent with previous studies of egg development and hatching success [23, 29]. Questing aggregations ranged vertically from 13–82 cm above ground level, with an average questing height of 56.8 cm (Fig. 4b), or generally the observed maximum height of available vegetation. Experimental studies have shown *D. albipictus* will preferentially aggregate at twice the maximum we observed, around 120 cm, or cervid host torso height [22]. Given ongoing infestation of Yukon cervids, vegetation height alone does not appear to be significantly limiting larval transmission to hosts in this system.

Understanding the location of the larval stage of *D. albipictus* may have important implications for future management of this species. Strategic burning of known larval hotspots has been proposed as a short-term control measure [30], or the development of known predators of larvae, such as entomopathogenic fungi, as a topically applied biological control agent [31]. Although there is little evidence that Yukon wildlife are currently adversely affected by tick infestation, the negative impact of *D. albipictus* hyperabundance elsewhere in North America suggests monitoring locations where larvae are found may provide options for proactive management or mitigation in future.

Our confirmed detection of *D. albipictus* larvae in Yukon using the flagging method represents a significant step in accumulating knowledge of this species *in-situ* in northern regions. All previous reported detections in Yukon and neighbouring Northwest Territories have been of adult and nymphal *D. albipictus*, either on-host [15, 16], or through anecdotal reports of potentially related hair loss on moose [13]. Detection of the off-host life stages of this tick provides evidence that suitable conditions exist for *D. albipictus* to complete its whole life cycle in Yukon, corroborating previous assertions of establishment potential [13, 28, 32], and provides critical information to inform ongoing monitoring and potential management or mitigation. Our detection may also forewarn of other tick species able to expand their range in the north, in line with a warming climate.

## Data Availability

The datasets used and/or analysed during the current study are available from the corresponding author on request.
